# Type of Dysmenorrhea, Menstrual Characteristics and Symptoms in Nursing Students in Southern Spain

**DOI:** 10.3390/healthcare8030302

**Published:** 2020-08-26

**Authors:** Ana Abreu-Sánchez, María Laura Parra-Fernández, María Dolores Onieva-Zafra, Juan Diego Ramos-Pichardo, Elia Fernández-Martínez

**Affiliations:** 1 Department of Nursing, University of Huelva, 21004 Huelva, Spain; abreu@denf.uhu.es (A.A.-S.); juan.ramos@denf.uhu.es (J.D.R.-P.); 2 Department of Nursing, Physiotherapy and Occupational Therapy, University of Castilla-La-Mancha, Ciudad Real, 13071 Ciudad Real, Spain; marialaura.parra@uclm.es (M.L.P.-F.); mariadolores.onieva@uclm.es (M.D.O.-Z.)

**Keywords:** dysmenorrhea, menstrual pain, symptom

## Abstract

Dysmenorrhea is a form of chronic pain associated with menstruation that affects a high percentage of young people. This study sought to determine the prevalence of primary and secondary dysmenorrhea among female nursing students in southern Spain and to compare their menstrual characteristics and symptoms. A cross-sectional observational study was conducted using a self-report questionnaire that included sociodemographic variables, menstrual characteristics and related symptoms. Descriptive bivariate analysis and binary logistic regression were performed in which the dependent variable was secondary dysmenorrhea. The prevalence of dysmenorrhea was 73.8% (of which 63.3% had primary dysmenorrhea and 10.5% had secondary dysmenorrhea), and was more likely in women with longer periods, heavier bleeding and those not using oral hormonal contraception (OCP). Secondary dysmenorrhea was 31.75, (confidence interval (CI)95% = 4.44–238.59; *p* < 0.01) times more likely among those with menstrual headache, 8.37 (CI95% = 2.35–19.80; *p* < 0.01), times greater among those suffering nausea during menstruation, 6.60 (CI95% = 1.42–30.67; *p* < 0.01), times greater among those suffering from menstrual vomiting, it was also 1.17 (CI95% = 1.08–1.26; *p* < 0.01) times more likely for each day that the period was prolonged and 6.63 (CI95% = 1.47–30.01; *p* = 0.014) times more likely in women with a heavy menstrual flow. These findings may help guide professionals towards the prescription of additional tests in certain cases in which secondary dysmenorrhea is suspected.

## 1. Introduction

A normal menstrual cycle is defined as a regular cycle ranging from 24 to 38 days, with an average blood loss of 5 to 80 mL, lasting 4.5 to 8 days [1,2]. Some women experience symptoms during menstruation that may condition their quality of life, especially among younger women. These include menstrual pain, nausea, vomiting, diarrhea, dizziness, irritability, depressive symptoms and headache. These symptoms, and menstrual pain in particular, can lead to absenteeism and reduced performance, and consequently have significant socioeconomic repercussions [3,4,5]. Evaluating women’s menstrual health is therefore a key aspect of health [6].

Menstrual pain or dysmenorrhea is a chronic, recurrent type of pain that manifests as menstrual cramps or painful periods, usually in the form of pelvic or lower abdominal pain, although it can also be experienced as low back pain and may be accompanied by other menstrual symptoms [7,8]. In relation to its physiopathology, two main types of dysmenorrhea are identified in the literature: primary and secondary dysmenorrhea. Primary dysmenorrhea is not associated with any known organic gynecological cause and has been traditionally associated with psychogenic causes, although it is currently more associated with biochemical causes, mainly an excess of prostaglandins and vasopressin [9,10,11]. In the case of secondary dysmenorrhea, the main cause is usually endometriosis, which is responsible for up to 62% of cases [12,13].

The clinical presentation of both types of dysmenorrhea is similar, as the main symptom is menstrual pain; however, some studies have identified specific symptoms related to certain gynecological pathologies such as pain during bowel movements and sexual intercourse among women who suffer secondary dysmenorrhea due to endometriosis [14]. Therefore, the differential diagnosis between both types of dysmenorrhea is not straightforward, as it is based on confirming or ruling out an organic cause related to dysmenorrhea in the case of suspected secondary dysmenorrhea [15,16,17]. However, previous studies have shown that most young people do not usually consult health professionals for menstrual pain, mostly due to feelings of shame and because this problem has become socially normalized [18,19]. This delay or absence of professional consultation implies a delay in the diagnosis, which, in the case of secondary dysmenorrhea, can lead to detection occurring at more advanced stages of the pathology, with a greater impact on women’s quality of life. In the case of primary dysmenorrhea, this also entails certain risks, although these are usually minor, such as those derived from self-medication for self-management of pain by young women and absenteeism from school and work due to ineffective self-management of pain [4,20]. Moreover, early physician consultation and interventions have been shown to be more cost-effective than self-care [21]. Nonetheless, even when professionals are consulted, complementary tests to rule out secondary dysmenorrhea are not usually performed.

The global prevalence of dysmenorrhea identified in previous studies varies, affecting approximately 70% of women of childbearing age [4]. In the Spanish population, there are few studies on this subject, with an estimated prevalence of dysmenorrhea of between 56–62% in the general population [22,23]. In the university population, primary dysmenorrhea was estimated at around 75% in a single study carried out at a university in central Spain [19]. No studies on the prevalence of dysmenorrhea and menstrual characteristics have been identified in the region of Andalusia, in southern Spain.

The present study aims to determine the prevalence of primary and secondary dysmenorrhea among university women in southern Spain and to compare their menstrual characteristics and symptoms.

The following hypothesis was proposed: the menstrual characteristics and symptoms of women with primary dysmenorrhea are different from those with secondary dysmenorrhea. Thus, the results of this study may serve to verify these characteristics and symptoms to guide health care providers in identifying women who require complementary testing to confirm secondary dysmenorrhea.

## 2. Materials and Methods

### 2.1. Design

A cross-sectional observational study was conducted in Huelva, Andalusia, an area in the southern region of Spain, among nursing students of the University of Huelva, between December 2019 and January 2020.

### 2.2. Participants and Sample

The inclusion criteria for the participants were women aged 18–35, who had seen a gynecologist at least once, enrolled in the Nursing Degree at the University of Huelva (Andalusia, Spain) during the 2019/2020 academic year, willingness to participate in the study and signing the informed consent. Students who were on an exchange at another university and who did not wish to participate were excluded.

All women who met the inclusion criteria were invited to participate and the study sample was created with the students who agreed to participate and who signed the informed consent (96.2%).

### 2.3. Study Variables and Data Collection

As there are no validated questionnaires available in Spanish, data were collected using an ad-hoc self-report questionnaire delivered on paper, which was purposely designed by the research team on the basis of previous studies [3,19,24,25]. The comprehension and content validity of the questionnaire was tested with a sample of 10 university students who did not participate in the study by making minor modifications after gathering the participants’ feedback. The questionnaire included sociodemographic and gynecological questions. Based on the definition of the types of dysmenorrhea [9,13], a woman was considered to have primary dysmenorrhea when she manifested menstrual pain but had not been diagnosed with any associated gynecological problem despite having consulted a doctor. Secondary dysmenorrhea was diagnosed when a woman suffered from menstrual pain and had been diagnosed with an organic gynecological problem in relation to the pain. Dichotomous questions were used to inquire about menstrual symptoms, in relation to the overall presence/absence of symptoms during periods, in the line of previous studies [3]. The Visual Analogue Scale (VAS) was used to evaluate the intensity of menstrual pain from 0 to 10, and the score was interpreted as in previous studies: mild (1–3), moderate (4–6) and severe (7–10) [19,26,27]. In the classroom setting, a teacher invited the students to participate in the study and a researcher provided information on the study aim.

### 2.4. Ethics

The participants voluntarily participated in the study and signed the informed consent form. This research project was conducted in accordance with the principles of the Helsinki Declaration. The Biomedical Research Ethics Committee of Andalusia granted ethical approval for this study prior to conducting the research (Ref. 4/20).

All collected data were processed anonymously in accordance with the current legislation on the protection of data.

### 2.5. Statistical Analysis

The collected data was entered onto a Microsoft Office Excel sheet for subsequent analysis using the Statistical Package for the Social Sciences (SPSS v23). For the descriptive analysis, the frequencies and percentages of the qualitative variables were used, whereas the means and standard deviations were calculated for quantitative variables.

To compare qualitative variables (sociodemographic and gynecological data) among women with and without dysmenorrhea, and among those with primary and secondary dysmenorrhea, the chi-square test was used. To compare quantitative variables between the same groups, the Student’s *t*-test was used. The Student’s *t*-test was also used to compare pain intensity using the VAS scale between different groups created according to the type of dysmenorrhea and in relation to the absence/presence of each menstrual symptom. The appropriateness of conditions for the application of the statistical tests employed was verified in all cases. A binary stepwise regression was performed, which only included the sample of participants with dysmenorrhea and was analyzed as a dependent variable in relation to suffering from secondary dysmenorrhea (yes/no). Sociodemographic characteristics, menstrual characteristics and symptoms were included as independent variables. The model was adjusted for age, age of menarche, body mass index (BMI), area of residence, consumption of oral hormonal contraception (OCP) and the intensity of menstrual pain variable (VAS). The significance level was *p* < 0.05.

## 3. Results

### 3.1. Sociodemographic and Gynecological Characteristics

The mean age of the 354 participants was 21.09 ± 2.40 years. Their BMI was 22.47 ± 3.27 kg/m^2^.

The mean age of menarche was 12.16 ± 1.54 years. Regarding the characteristics of the menstrual cycle, the mean number of days per cycle was 29.84 ± 8.07 and the length of menses (days of bleeding) was 4.97 ± 1.25 days. Up to 70.3% of women reported a regular cycle and 61% had a moderate amount of menstrual flow.

In total, 64.4% of women did not use any form of contraception. Among the women who did use contraception, 88.8% used the combined pill, 8% used the vaginal ring, 2.4% reported using the intrauterine device and 0.8% used the transdermal patch.

The prevalence of dysmenorrhea was 73.8% (261), of which 85.8% (224) corresponded to primary dysmenorrhea and 14.2% (37) to secondary dysmenorrhea. Among the latter group, endometriosis was reported in 24 students (64.9%), followed by polycystic ovary syndrome (10 students, 27.0%). Table 1 compares the sociodemographic and gynecological variables between women in relation to whether they suffer from dysmenorrhea and the type of dysmenorrhea they suffer.

### 3.2. Location of Menstrual Pain

As reflected in Table 2, 81.2% of women with dysmenorrhea suffered from pain in the pelvic area or lower abdominal area (81.2%), both for women with primary dysmenorrhea and for women with secondary dysmenorrhea. The second most frequent location was low back pain (42.1%), also for both types of dysmenorrhea. No differences were found between the location of menstrual pain and the type of dysmenorrhea (*p* > 0.05).

### 3.3. Symptoms Associated with Menstruation

As shown in Table 3, up to 87.6% participants reported fatigue, 67.8% felt depressed, 61% suffered from diarrhea and 57.3% reported headaches. Irritability was reported in 36.4% of participants, whereas 30.2% suffered from nausea, dizziness affected 27.7%, constipation affected 12.4% and 8.8% suffered from vomiting. In women with dysmenorrhea, the most common symptoms were fatigue (87.8%), depressive symptoms (69.3%), diarrhea (61.2%) and headache (57.5%). Dizziness (*p* = 0.000), nausea (*p* = 0.001), vomiting (*p* = 0.009) and fatigue (*p* = 0.001) were more frequent in women with dysmenorrhea compared to those without dysmenorrhea. The comparison of menstrual symptoms between women with primary and secondary dysmenorrhea revealed statistically significant differences in irritability (*p* = 0.015) and nausea (*p* = 0.038), which was more common in women with secondary dysmenorrhea. 

Regarding the intensity of menstrual pain measured using the VAS, no difference was found between the mean scores of women with both types of dysmenorrhea. The mean score in women with primary dysmenorrhea was 7.30 ± 1.47 whereas in women with secondary dysmenorrhea, this was 6.88 ± 1.78 (*p* > 0.05).

Table 4 shows that women who experienced either dizziness, headache, depressive symptoms, diarrhea, nausea, vomiting, and fatigue as a menstrual symptom had higher menstrual pain intensity scores than those who did not. 

### 3.4. Multivariate Regression: Secondary Dysmenorrhea and Menstrual Characteristics and Symptoms

The results of the multivariate regression are summarized in Table 5. In woman with dysmenorrhea, secondary dysmenorrhea was 31.75 (CI95% = 4.44–238.59; *p* < 0.01) times more likely among those with menstrual headache, 8.37 (CI95% = 2.35–19.80; *p* < 0.01), times greater in women with nausea during menstruation, 6.60 (CI95% = 1.42–30.67, *p* < 0.01) times higher among those suffering from menstrual vomiting, 1.17 (CI95% = 1.08–1.26; *p* < 0.01) times more likely for each day that the period was extended and 6.63 (CI95%1.47–30.01; *p* = 0.014) times more likely in women with a heavy menstrual flow. 

## 4. Discussion

The aim of the present study was to determine the prevalence of primary and secondary dysmenorrhea among university women in southern Spain, and to compare their menstrual characteristics and symptoms. We identified a prevalence rate of 73.8% for dysmenorrhea (of which 63.3% was primary dysmenorrhea, and 10.5% was secondary dysmenorrhea). Dysmenorrhea was more frequent in women with a longer length of menses, in those with heavier periods, and women not using OCPs. In terms of menstrual symptoms, fatigue, nausea and vomiting were more common among young women with menstrual pain than in those without; in addition, young women suffering from dizziness, headache, depressive symptoms, diarrhea, nausea, vomiting and fatigue during menstruation had higher mean scores for intensity of menstrual pain measured using the VAS. The results of multivariate regression showed that women with menstrual pain were more likely to have secondary dysmenorrhea than a greater volume of menstrual flow, more days between periods, greater menstrual nausea and vomiting, a greater incidence of menstrual headache and greater use of OCPs than those with primary dysmenorrhea. The location of the pain, however, was similar in both types of dysmenorrhea.

The prevalence of dysmenorrhea in our sample is similar to that reported in the systematic review and meta-analysis carried out by Armour et al. based on international studies [4]. According to our findings, 63.3% of university women in southern Spain suffer from dysmenorrhea, a percentage similar to a study by Ortiz et al. (64%) among Mexican university women [24]. However, this is lower than the prevalence reported in the Spanish region of Ciudad Real (74.8%), the only study on this subject conducted among Spanish university students [19]. Compared to European studies, the prevalence was lower than a study carried out on Greek nursing students which identified 89.2% of young people with dysmenorrhea [28] and lower than a prevalence of 89% identified in young Swedes [29], although similar to the 62.8% identified in Portuguese women [30]. These differences among European countries may be related to differences affecting the climate, lifestyle and diet. Nonetheless, the lifestyle is more similar between Spain and Portugal; hence, perhaps this explains the similar prevalence between both countries. In addition, it is worth considering that the region where this study was conducted receives more hours of sunlight than the rest of Spain (indeed, this region is known as “the coast of light”). Consequently, one can assume that the vitamin D of these young women is higher than that of the rest of Spain, understanding that vitamin D levels have been associated with a lower incidence of menstrual disorders and dysmenorrhea [31,32].

The finding that the frequency of dysmenorrhea was higher in young women who experienced longer periods and greater menstrual bleeding was consistent with previous studies in other countries and in Spain [9,19]. Furthermore, women with secondary dysmenorrhea experienced more days of menstrual pain than those with primary dysmenorrhea. This may be because the main cause of secondary dysmenorrhea is endometriosis and women with this condition tend to experience more days of pain per month [13]. However, the fact that women with secondary dysmenorrhea have longer periods and greater flow is striking since these women consume the most OCPs and could therefore be expected to have shorter periods.

We found that the frequency of dysmenorrhea was higher in young women with more prolonged menstrual periods and with a heavier menstrual flow, which was in line with previous studies conducted in other countries and in Spain [9,19]. Women with secondary dysmenorrhea experienced more days of menstrual pain than those with primary dysmenorrhea. This may be because the main cause of secondary dysmenorrhea was endometriosis and in women with this pathology, there are usually more days of pain per month [13]. However, the fact that women with secondary dysmenorrhea have longer menses is striking, as they are the ones who use the most OCPs and could therefore be expected to have less menstrual bleeding. Therefore, in future studies it would be interesting to examine the origin of secondary dysmenorrhea in each participant and explore this issue.

Regarding the consumption of OCPs, the finding that there was a higher proportion of women with dysmenorrhea among those not using OCPs is consistent with previous studies that indicated the effectiveness of OCPs against dysmenorrhea [33,34]. In addition, this study identified that women with secondary dysmenorrhea used more OCPs than those with primary dysmenorrhea, which is consistent with the therapeutic approach for both types of dysmenorrhea. Thus, in the case of primary dysmenorrhea, the first line treatment is non-steroidal anti-inflammatory drugs, however, for secondary dysmenorrhea a more specific approach targeted at the cause of menstrual pain is required [35]. Bearing in mind that the most common cause of secondary dysmenorrhea is endometriosis, the most common pharmacological approach is the consumption of OCPs [12,36].

All of the menstrual symptoms were more frequent in women suffering a greater intensity of menstrual pain, as reported in the national study conducted by Zhao Hu et al. among Chinese university women [37]. This is consistent with the authors indicating that these symptoms are attributed to systemic pain symptoms [9]. However, it is noteworthy that many women without dysmenorrhea also suffer from these symptoms. In our study, we found that only dizziness, nausea, vomiting and fatigue were statistically more frequent in women with dysmenorrhea. Thus, when conducting a menstrual health assessment, all women should be questioned about these symptoms and not only women with dysmenorrhea. In addition, specific approaches to symptoms should be explored to avoid focusing exclusively on the management of dysmenorrhea pain. In this line, in recent years, the effectiveness of certain non-drug methods, such as ginger, has been demonstrated for the relief of both menstrual pain and menstrual nausea [38].

In relation to menstrual symptoms, the main findings of our study indicate that nausea, vomiting and headache were more likely in those with secondary dysmenorrhea compared to other women with menstrual pain. The results for gastrointestinal symptoms are consistent with those reported by Malin et al. in Swedish women and DiVasta et al. in American women [14,39], which attribute these findings to hormonal implications. However, other studies such as the Evans et al. found no difference in the symptoms of women with primary dysmenorrhea and women diagnosed with secondary dysmenorrhea [40]. The relationship between menstrual headache and organic pathologies that produce secondary dysmenorrhea, such as endometriosis, has been described in the last decade in several studies [41,42,43]; however, its physiopathological relationship, which is usually attributed to a hormonal influence, is unclear [44]. It should be noted that treatment with OCPs is most common in women with secondary dysmenorrhea, and therefore headaches may not be so much related to the pathology, but rather reflect a proven side effect of the treatment [45]. In our participants, nausea and irritability were also statistically more frequent in women with secondary dysmenorrhea compared to those with primary dysmenorrhea, although these variables did not enter the final regression model. This may be due to the fact that the diagnosis of the gynecological problems that produce this type of chronic pelvic pain tend to be delayed, leading to more chronic conditions, often associated with the development of mental health problems and a decline in quality of life [46,47,48]. In addition, the principal treatment for these disorders is usually hormonal, which may also be influencing mood and therefore the frequency of symptoms [48,49].

The strengths of this study are that it confirms that dysmenorrhea affects a large number of young people in southern Spain. Furthermore, certain characteristics and symptoms have been identified as being more frequent in women with secondary dysmenorrhea than in those with primary dysmenorrhea. This may make it easier to identify women who potentially have this problem in a clinical interview. If these results are corroborated in multicenter studies with larger sample sizes, it could be very useful to optimize resources by prescribing tests for the differential diagnosis of the type of dysmenorrhea among women with certain specific profiles. Another noteworthy finding is that we have identified that both women with and without dysmenorrhea often display a variety of menstrual symptoms, suggesting a need for greater awareness in this regard, both among professionals and the general population. In addition, having identified that menstrual symptoms are more frequent in women with more severe menstrual pain supports the fact that health professionals should be prepared to offer advice not only on menstrual pain but also to explore and know how to provide advice on methods to relieve other menstrual symptoms. Concerning the limitations of this study it should be noted that the data were obtained based on a cross-sectional design and that participants were drawn from a single college and university. Another limitation was that the diagnosis of primary and secondary dysmenorrhea was self-reported by the patients in the data collection questionnaire, based on the diagnosis by a gynecologist, considering that the inclusion criterion stated that participants must have visited the gynecologist at least once. However, in the context of the study, no discriminatory medical tests were performed. This may be of interest for inclusion in future studies.

## 5. Conclusions

The prevalence of dysmenorrhea is high among young women in Andalusia, southern Spain. A higher incidence was found in women with longer periods, a heavier menstrual flow and women not using OCPs. These characteristics vary in relation to the type of dysmenorrhea. Thus, women with secondary dysmenorrhea had a greater amount of menstrual flow and experienced more days of menstrual pain. Women with menstrual pain were more likely to have secondary dysmenorrhea if they had heavy amounts of bleeding, longer intervals between periods, and three key symptoms: the presence of headache, nausea, and vomiting. These gynecological characteristics can guide us in identifying women who are more likely to have primary dysmenorrhea once secondary dysmenorrhea has been ruled out. In these cases, education in pain self-management may be provided, in order to decrease the prevalence of other menstrual symptoms. In addition, by identifying which characteristics are more common in women with secondary dysmenorrhea, this can support the request for further complementary gynecological diagnostic tests in women with menstrual pain to confirm the etiology of the symptoms, before assuming primary dysmenorrhea. Further research along this line may allow us to optimize health resources and reduce the delay in the diagnosis of the etiology of secondary dysmenorrhea, thus enhancing the quality of life of women suffering from these symptoms.

## Figures and Tables

**Table 1:** Comparison of sociodemographic and gynecological variables between women in relation to whether they suffer from dysmenorrhea and the type of dysmenorrhea.

		Dysmenorrhea (*n* = 354)		Total M ± SD/n(%)	*p*-Value	Type of Dysmenorrhea (*n* = 261)		Total M ± SD/n(%)	*p*-Value
		No (*n* = 93) M ± SD/ n(%)	Yes (*n* = 261) M ± SD/ n(%)			Primary (*n* = 224) M ± SD/ n(%)	Secondary (*n* = 37) M ± SD/ n(%)		
Age (years)		21.33 ± 2.66	21.00 ± 2.29	21.09 ± 2.40	0.250	20.96 ± 2.24	21.24 ± 2.64	21.00 ± 2.29	0.487
Residence	Urban	72(77.4%)	200(76.6%)	271(76.8%)	0.877	168(75%)	32(86.5%)	200(76.6%)	0.126
	Rural	21(22.6%)	61(23.4%)	82(23.2%)		56(91.8%)	5(13.5%)	61(23.4%)	
BMI		22.53 ± 3.02	22.44 ± 3.36	22.47 ± 3.27	0.819	22.31 ± 3.25	23.20 ± 3.88	22.44 ± 3.36	0.134
Age of menarche		12.18 ± 1.52	12.15 ± 1.55	12.16 ± 1.54	0.858	12.15 ± 1.52	12.16 ± 1.78	12.15 ± 1.55	0.957
Days between periods		30.95 ± 12.47	29.45 ± 5.73	29.84 ± 8.07	0.267	29.05 ± 4.46	31.89 ± 10.34	29.45 ± 5.73	0.108
Menses length, days		4.63 ± 1.21	5.08 ± 1.25	4.97 ± 1.25	0.003 *	5.03 ± 1.26	5.43 ± 1.14	5.08 ± 1.25	0.067
Amount of menstrual flow (ml)	Light (≤5 pads/day)	28(30.1%)	34(13%)	62(17.5%)	0.000 *	30(13.4%)	4(10.8%)	34(13%)	0.026 *
	Medium (5–7 pads/day)	61(65.6%)	155(59.4%)	216(61%)		139(62.1%)	16(43.2%)	155(59.4%)	
	Heavy (≥7 pads/day)	4(4.3%)	72(27.6%)	76(21.5%)		55(24.6%)	17(45.9%)	72(27.6%)	
Regular	No	29(31.2%)	76(29.1%)	105(29.7%)	0.708	61(27.2%)	15(40.5%)	76(29.1%)	0.099
	Yes	64(68.8%)	185(70.9%)	249(70.3%)		163(72.8%)	22(59.5%)	185(70.9%)	
OCP	No	39(41.9%)	189(72.4%)	228(64.4%)	0.000 *	172(76.8%)	17(45.9%)	189(72.4%)	0.000 *
	Yes	54(58.1%)	72(27.6%)	126(35.6%)		52(23.2%)	20(54.1%)	72(27.6%)	
Days of menstrual pain		-	-	-	-	2.34 ± 1.05	2.86 ± 1.29	2.42 ± 1.10	0.007 *

BMI (Body Mass Index); OCP (oral hormonal contraception); M (Mean); SD (Standard deviation); * *p* < 0.05.

**Table 2:** Location of menstrual pain: primary and secondary dysmenorrhea.

		Type of Dysmenorrhea		Total	*p*-Value
		Primary	Secondary		
Pelvic or lower abdominal pain	No	41(18.3%)	8(21.6%)	49(18.8%)	0.632
	Yes	183(81.7%)	29(78.4%)	212(81.2%)	
Low back pain	No	130(58%)	21(56.8%)	151(57.9%)	0.884
	Yes	94(42%)	16(43.2%)	110(42.1%)	
Lumbosacral pain	No	185(70.9%)	27(73%)	212(81.2%)	0.165
	Yes	39(14.9%)	10(27%)	49(18.8%)	
Genital pain	No	158(70.5%)	24(64.9%)	182(69.7%)	0.487
	Yes	66(29.5%)	13(35.1%)	79(30.3%)	

**Table 3:** Symptoms associated with menstruation in women with and without dysmenorrhea.

		Dysmenorrhea (*n* = 354)		Total	*p*-Value	Type of Dysmenorrhea (*n* = 261)		Total	*p*-Value
		No	Yes			Primary	Secondary		
Dizziness	No	80(87%)	175(67%)	255(72.2%)	0.000 *	153(68.3%)	22(59.5%)	175(67%)	0.289
	Yes	12(3.4%)	86(33%)	98(27.8%)		71(31.7%)	15(40.5%)	86(33%)	
Headache	No	52(56.5%)	98(37.5%)	150(42.5%)	0.155	88(39.3%)	10(10.2%)	98(37.5%)	0.154
	Yes	40(43.5%)	163(62.5%)	203(57.5%)		136(60.7%)	27(73%)	163(62.5%)	
Irritability	No	65(69.9%)	160(61.3%)	225(63.6%)	0.139	144(64.3%)	16(43.2%)	160(61.3%)	0.015 *
	Yes	28(30.1%)	101(38.7%)	129(36.4%)		80(35.7%)	21(56.8%)	101(38.7%)	
Depressive symptoms	No	34(36.6%)	80(30.7%)	114(32.2%)	0.295	69(30.8%)	11(29.7%)	80(30.7%)	0.896
	Yes	59(63.4%)	181(69.3%)	240(67.8%)		155(69.2%)	26(70.3%)	181(69.3%)	
Diarrhea	No	41(44.6%)	96(36.8%)	137(38.8%)	0.188	85(37.9%)	11(29.7%)	96(36.8%)	0.337
	Yes	51(23.6%)	165(63.2%)	216(61.2%)		139(62.1%)	26(70.3%)	165(63.2%)	
Constipation	No	83(90.2%)	226(86.6%)	309(87.5%)	0.365	192(85.7%)	34(91.9%)	226(86.6%)	0.307
	Yes	9(9.8%)	35(13.4%)	44(12.5%)		32(14.3%)	3(8.1%)	35(13.4%)	
Nausea	No	79(85.9%)	168(64.37%)	247(69.77%)	0.001 *	148(69.08%)	20(54.05%)	168(64.37%)	0.038 *
	Yes	13(14.1%)	93(35.63%)	107(30.23%)		76(29.92%)	17(45.95%)	93(35.63%)	
Vomiting	No	91(97.8%)	232(88.9%)	323(91.2%)	0.009 *	200(89.3%)	32(86.5%)	232(88.9%)	0.616
	Yes	2(2.2%)	29(11.1%)	31(8.8%)		24(10.7%)	5(13.5%)	29(11.1%)	
Breast pain	No	55(98.2%)	251(97.7%)	306(97.8%)	0.801	35(94.6%)	216(98.2%)	251(97.7%)	0.181
	Yes	1(1.8%)	6(2.3%)	7(2.2%)		2(5.4%)	4(1.6%)	6(2.3%)	
Fatigue	No	20(21.7%)	23(8.8%)	43(12.2%)	0.001 *	21(9.4%)	2(5.4%)	23(8.8%)	0.430
	Yes	72(78.3%)	238(91.2%)	310(87.8%)		203(90.6%)	35(94.6%)	238(91.2%)	

* *p* < 0.05.

**Table 4:** Intensity of menstrual pain in women with dysmenorrhea and menstrual symptoms.

		Intensity of Menstrual Pain VAS		*p*-Value
		Media	DS	
Dizziness	No	6.28	1.93	0.000 *
	Yes	7.36	1.65	
Headache	No	6.11	1.99	0.000 *
	Yes	6.94	1.78	
Irritability	No	6.46	2.04	0.075
	Yes	6.84	1.67	
Depressive symptoms	No	6.10	2.20	0.002 *
	Yes	6.85	1.71	
Diarrhea	No	6.31	2.15	0.032 *
	Yes	6.78	1.74	
Constipation	No	6.57	1.92	0.431
	Yes	6.82	1.88	
Nausea	No	6.25	1.92	0.000 *
	Yes	7.44	1.60	
Vomiting	No	6.44	1.91	0.000 *
	Yes	8.22	1.84	
Breast pain	No	6.67	1.80	0.116
	Yes	7.83	1.72	
Fatigue	No	5.31	1.98	0.000 *
	Yes	6.79	1.85	
* *p* < 0.05				

**Table 5:** Multivariate regression: secondary dysmenorrhea and menstrual characteristics and symptoms.

	OR ^a^	CI95%	*p*-Value
Headache	31.75	4.44–238.59	0.001 **
Nausea	8.37	2.35–19.80	0.001 **
Vomiting	6.60	1.42–30.67	0.001 **
Days between periods	1.17	1.08–1.26	0.000 **
Amount of menstrual flow Heavy (≥7 pads/day)	6.63	1.47–30.01	0.014 *

^a^ Adjusted for age, age of menarche, BMI, area of residence, OCP consumption and pain intensity (VAS); * *p* < 0.05; ** *p* < 0.01.
